# Strategies Used by SARS-CoV-2 to Evade the Innate Immune System in an Evolutionary Perspective

**DOI:** 10.3390/pathogens13121117

**Published:** 2024-12-17

**Authors:** Hong Fan, Mingfu Tian, Siyu Liu, Chenglin Ye, Zhiqiang Li, Kailang Wu, Chengliang Zhu

**Affiliations:** 1 Department of Clinical Laboratory, Institute of Translational Medicine, Renmin Hospital of Wuhan University, Wuhan 430060, China; 2023203020025@whu.edu.cn (H.F.); 2019283020200@whu.edu.cn (C.Y.); lizhq2022@whu.edu.cn (Z.L.); 2 State Key Laboratory of Virology, College of Life Sciences, Wuhan University, Wuhan 430072, China; 2015202040056@whu.edu.cn (M.T.); 2020202040047@whu.edu.cn (S.L.); wukailang@whu.edu.cn (K.W.)

**Keywords:** SARS-CoV-2, innate immune evasion, interferon, Omicron

## Abstract

By the end of 2019, the COVID-19 pandemic, resulting from the Severe Acute Respiratory Syndrome Coronavirus 2 (SARS-CoV-2), had diffused widely across the globe, with 770 million infected individuals and over 7 million deaths reported. In addition to its high infectivity and pathogenicity and its rapid mutation rate, the unique capacity of SARS-CoV-2 to circumvent the immune system has also contributed to the widespread nature of this pandemic. SARS-CoV-2 elicits the onset of innate immune system activation and initiates antiviral responses once it has infected the host. While battling the host’s immune responses, SARS-CoV-2 has established many countermeasures to evade attack and clearance. As the exploration of SARS-CoV-2 continues, substantial evidence has revealed that the 29 proteins synthesized by the SARS-CoV-2 genome are integral to the viral infection process. They not only facilitate viral replication and transmission, but also assist SARS-CoV-2 in escaping the host’s immune defenses, positioning them as promising therapeutic targets that have attracted considerable attention in recent studies. This review summarizes the manner in which SARS-CoV-2 interfaces with the innate immune system, with a particular focus on the continuous evolution of SARS-CoV-2 and the implications of mutations.

## 1. Introduction

**Figure 1:**
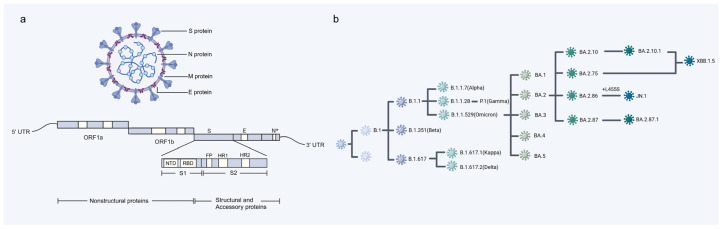
SARS-CoV-2 structure and evolution. (**a**) Schematic representation of the genome of SARS-CoV-2. (**b**) Phylogenetic tree of SARS-CoV-2 VOCs and Omicron sublineages. ‘*’ is used to emphasize that this region has significant importance in gene structure or function.

Like other coronaviruses, SARS-CoV-2 is a positive-sense single-stranded RNA virus [1]. ORF1a and ORF1b are located at the 5′ end of the genome encode polypeptide pp1a and pp1b, which are processed by virus proteases NSP3 containing a papain-like protease domain and NSP5 containing a 3C-like protease domain, generating 16 nonstructural proteins, while genome 3′s end encodes structural protein (Figure 1a) [2,3].

When the spike protein on the envelope of SARS-CoV-2 binds to the cell membrane receptor angiotensin-converting enzyme 2 (ACE2), SARS-CoV-2 enters the cell through receptor-mediated endocytosis, which prompts the host immune to respond. The host rapidly recognizes and responds to viral infections through the innate immune system, making innate immunity the cornerstone of defense against viral invasion. Pattern recognition receptors (PRRs) located on cell membranes, endosomal membranes, lysosomal membranes, and in the cytoplasm detect the invasion of viral pathogen-associated molecular patterns (PAMPs) or damage-associated molecular patterns (DAMPs) such as exposed mitochondrial DNA, initiating signaling pathways for host inflammation and antiviral immune responses, thereby protecting the host from infection [4]. As an essential component of innate immunity, interferons (IFNs) transmit signals via the JAK-STAT pathway upon receptor binding, activating downstream signaling cascades and transcription factors and inducing hundreds of IFN-stimulated genes (ISGs). ISGs and their expression products can resist viral infection by inhibiting virus replication. [5]. SARS-CoV-2 utilizes multiple strategies to evade host innate immune system attacks, allowing significant viral replication and favoring prolonged infection [6,7].

In the process of the rapid global spread of SARS-CoV-2, its large genome underwent frequent mutations, particularly in the receptor-binding domain (RBD) of the spike protein. These mutations are pivotal to the evolutionary adaptation of SARS-CoV-2, enabling strains with these mutations to swiftly supersede the original strain as the predominant source of infection [8]. By the conclusion of 2020, numerous variants with increased transmissibility, heightened pathogenicity, or partial immune escape emerged, becoming classified as variants of concern (VOCs) [9,10]. The last variant in this category was Omicron, which was designated as a VOC in November 2021. Since then, most circulating variants have been sublineages of Omicron, and they are primarily monitored as variants under monitoring (VUMs) or variants of interest (VOIs) due to their impact on transmission, immune escape, and disease severity (Figure 1b) [11]. In this review, the critical role of SARS-CoV-2 proteins in evading innate immunity and how each protein targets specific signaling pathways and their mechanisms of interfering with the immune response are highlighted from multiple perspectives.

## 2. SARS-CoV-2 Infection and Innate Immunity

RIG-I-like receptor (RLR) signaling represents a cornerstone of the innate immune system to resist RNA virus infection. The human genome encodes three RLRs, namely Retinoic Acid-Inducible Gene I (RIG-I), Melanoma Differentiation-Associated Protein 5 (MDA5), and Laboratory of genetics and physiology 2 (LGP2), belonging to the family of RNA helicases structurally possessing DexH-conserved sequences [12].

RIG-I is non-functional at rest, with its CARDs domain bound to the insertion region of the helicase domain. Following the detection of viral RNA by RIG-I, and specific binding of viral RNA structures through its C-terminal RBD, RIG-I molecules transition to an open conformation, exposing the N-terminal signaling domain (CARD), recruiting E3 ubiquitin ligases such as TRIM25, catalyzing K63 ubiquitination modification of RIG-I, and subsequently binding to link Mitochondrial antiviral signaling protein (MAVS), leading to the phosphorylation of interferon regulatory factor 3/7 (IRF3/7). Phosphorylated and dimerized IRF3/7 translocate to the nucleus, promoting the production of IFN-α/β [13].

The Toll-like receptor (TLR) family, comprising 10 families in the human body, is another major innate immune receptor. TLR1, TLR2, TLR4, TLR5, TLR6, and TLR10 are located on the cell membrane, while the remaining are intracellular and exist in the endoplasmic reticulum (ER), endosomes, or lysosomes [14]. The signaling of TLRs requires the involvement of various adapter molecules, including Myeloid differentiation primary response 88 (MyD88), TIR-domain-containing adapter-inducing interferon-β (TRIF), TIR-domain-containing adapter protein (TIRAP)/MyD88 adapter-like (MAL), and TRIF-related adapter molecule (TRAM), which bind to the TIR domain and transmit inflammatory signals. Apart from TLR3, the rest of the TLRs require the participation of MyD88 in recognizing corresponding pathogen-associated molecular patterns [15]. Following this recognition, these TLRs form dimers, such as homodimers for TLR4 and heterodimers for TLR2 and TLR1. Upon dimerization, the TLRs recruit MyD88 through their TIR domains, activate IRAK4, and phosphorylate IRAK1, and the activation of these kinases is transmitted through TNF-receptor-associated factor 6 (TRAF6) to activate downstream molecule nuclear factor-κB (NF-κB). In contrast, TLR3 can simultaneously activate both the IRF3/7 and NF-κB pathways simultaneously via the TRIF pathway and induce the creation of type I interferons and inflammatory factors [16].

It is noteworthy that the activation of TLRs is a double-edged sword. Although the activation of TLRs can rapidly initiate the innate immunity in the early stage of infection, the continuous activation of TLRs may lead to serious problems [17]. In the late stage of SARS-CoV-2 infection, the virus replication begins to decline and a large number of immune cells are recruited to the lungs. A recent study has found that immune cells recognize and bind to the structural proteins E and M of SARS-CoV-2 through the highly expressed TLR1 receptor on the surface, triggering the activation of a violent inflammatory response. Meanwhile, SARS-CoV-2 combined with TLR1 can be taken into the immune cells through endocytosis. This "abortive infection" pattern will prevent SARS-CoV-2 from forming the replication and transcription complex (RTC) to transcribe the subgenomic RNA of SARS-CoV-2. Due to the lack of subgenomic RNA, the structural proteins of SARS-CoV-2 and the accessory proteins of SARS-CoV-2 including Orf6 cannot be expressed, but the pro-inflammatory protein Nsp14 can be directly translated from the genomic RNA. Because only Nsp14 is expressed without the expression of Orf6, the inflammatory response is further amplified, and immune cells release a large quantity of inflammatory factors, resulting in an inflammatory cytokine storm [18].

Upon the union of the S protein of the SARS-CoV-2 to the ACE2 receptor, the cells undergo fusion, resulting in syncytia formation and subsequent chromosomal damage. This process releases nuclear DNA, activating the cytoplasmic double-stranded DNA sensor, cyclic GMP-AMP (cGAMP) synthase (cGAS) [19,20]. When DNA is sensed and bound by cGAS, it orchestrates a biochemical alteration, catalyzing the fabrication of a dimeric nucleotide, cGAMP, which serves as a critical second messenger. cGAMP binds to the endoplasmic reticulum (ER)-located cyclic dinucleotide sensor STING, triggering STING oligomerization. Following oligomerization, STING is trafficked from the ER to the Golgi apparatus, at which point it acts to recruit and activate TANK-binding kinase 1 (TBK1) using the ER-Golgi intermediate compartment (ERGIC) and the Golgi apparatus [21]. TBK1 then acts as a kinase, modifying itself, STING, and later the IRF3 transcription factor, causing IRF3 dimerization and translocation into the nucleus, stimulating the manufacturing of type I IFNs and the enactment of interferon-stimulated genes, collectively coordinating antiviral defense mechanisms [22]. 

After the virus-induced secretion of type I IFNs, these molecules bind to the type I IFN receptor complex (IFNAR1 and IFNAR2), and this interaction culminates in the activation of Janus kinase 1(JAK1) and tyrosine kinase 2(TYK2). JAK-mediated tyrosine phosphorylation activates the signal transducer and the activator of transcription 1 (STAT1) and STAT2. In conjunction with the DNA-binding component IRF9, they constitute a trimeric complex designated interferon-stimulated gene factor 3 (ISGF3). ISGF3 is subsequently transferred to the nucleus, within which it binds to interferon-stimulated response elements (ISREs) on the transcription start sites of numerous ISGs, thereby activating their transcription. ISG-encoded proteins generate efficacious antiviral innate immunity to clear invading viruses [23].

## 3. The Strategy of SARS-CoV-2 to Evade Innate Immune Response

### 3.1. Nsp15 Helps SARS-CoV-2 Evade the Recognition of PRRs

Cells infected with SARS-CoV-2 exhibit the assembly of stress granules (SGs). G3BP is a core protein of SG formation in this process, its isoform G3BP1 promoting the binding of mtDNA to cGAS [24]. A recent study found that the N protein was capable of forming aggregates within cells and recruit G3BP1, thereby diminishing the auxiliary role of G3BP1 in cGAS. However, whether these processes occur in precisely the same manner within infected cells remains to be further investigated [25]. Nsp15 functions as an endoribonuclease that cleaves 5′-polyuridines from the minus strand of viral RNA, thus reducing the deposition of viral PAMPs and evading recognition by PRRs [26,27]. During replication, coronaviruses produce double-stranded RNA (dsRNA) intermediates, enabling the dsRNA sensor MAVS to recognize them. Nsp15 likely disrupts dsRNA sensors that promote IFN expression, serving as a potent mediator of viral immune evasion [28,29].

### 3.2. Proteins That Help SARS-CoV-2 Inhibit Type I IFN Production

#### 3.2.1. N Protein, Nsp5, and PLpro Reduce Activation of RIG-I/MDA5

The activity of RIG-I is inhibited in uninfected cells, and its activation is contingent upon the E3 ubiquitin ligase properties of TRIM25 [30]. SARS-CoV-2 N protein and Nsp5 competitively bind to TRIM25, which in turn results in the hindrance of RIG-I ubiquitination and the subsequent suppression of downstream signaling pathways [31]. Type I interferon induces the secretion of ISG15, which is structurally homologous to ubiquitin and falls within the ubiquitin-like protein family (Ubls). ISGylation of MDA5 is a vital step in the stimulation of downstream signaling pathways following a viral infection [32]. SARS-CoV-2 PLpro engages with ISG15 through its “thumb” subdomain, causing the Ubl to be unwound from the “fingers” subdomain, and thus impeding ISG15-dependent MDA5 activation and the inhibition of host cell antiviral responses [33].

#### 3.2.2. Orf9b and Nsp5 Affect the Activity of MAVS

TOM70, a member of the translocase of the outer membrane (TOM) complex family, is indispensable for the activation of MAVS [34]. SARS-CoV-2 Orf9b has the capacity to exert an inhibitory effect on the interaction between the TOM70 and HSP90 proteins by binding to TOM70 [35,36]. It has been evidenced that the full-length form of HSP90 is in a position to interact and function with free TOM70. However, it is unable to bind to the ORF9b-Tom70 complex [37]. As a protease, Nsp5 cleaves the N-terminal amino acids of RIG-I, rendering it incapable of activating MAVS. Furthermore, it promotes MAVS ubiquitination at the K136 residue through E3 ubiquitin ligase activity, targeting K48-linked polyubiquitin molecules to MAVS for proteasome-mediated degradation [38,39].

#### 3.2.3. S Protein, M Protein, Orf8, Nsp5, and Nsp13 Block the Nuclear Localization of IRF3

SARS-CoV-2 S protein likely specifically targets type I interferon responses rather than pro-inflammatory signaling responses; mechanistically, S protein exerts its effect by facilitating the proteasome-mediated degradation of IRF3 [40]. The M protein interacts with the nuclear transport protein KPNA6 to hinder the nuclear translocation of IRF3, thereby reducing IFN production. It is conceivable that a segment of the M protein may attach to the nuclear membrane surface, blocking the association between KPNA6 and IRF3 within the nucleus [41]. However, these conclusions are all drawn based on the overexpression system. A potential interaction between Orf8 and HSP90B1 reveals that Orf8 significantly reduced the nuclear translocation of IRF3 and suppressed the type I interferon response [42,43,44]. Nsp13 binds to the IAD structural domain of IRF3 via the 1B structural domain, thereby disrupting IRF3-directed signal transduction [45]. In the downstream phase, it has been proven that the Nsp5 of SARS-CoV-2 can suppress the production of IFN induced by the Sendai virus, that its mechanism is to prevent the nuclear translocation of phosphorylated IRF3 [46,47,48]. At present, it is believed that the C-terminal of Orf3b, one of the lowest expression proteins of SARS-CoV-2, contains a nuclear localization signal (NLS) and weakens the ability of IRF3 to translocate into the nucleus antagonizing the function of IFN [49,50]. 

#### 3.2.4. M Protein and Nsp13 Target TBK1

It is hypothesized that the SARS-CoV-2 M protein may degrade the TBK1 protein through K48 ubiquitination [51]. Sequestosome 1 (SQSTM1), also known as p62, is a multifunctional protein that is involved in various cellular processes, including autophagy and the regulation of signaling pathways [52]. Nsp13 overexpression has been observed to reduce p62 expression while simultaneously inducing p62 aggregation and recruiting TBK1 to form a complex. In this process, the Rec A domain of Nsp13 has been shown to interact with TBK1, thereby inhibiting the phosphorylation of TBK1. Conversely, p62 has been demonstrated to bind primarily to the ZBD domain of Nsp13. This complex has been identified as targeting autophagosomes and subsequently degrading TBK1 following fusion with lysosomes [52].

#### 3.2.5. Orf9b Interferes with the Activation of NF-κB

In addition to suppressing the recognition of viral RNA by MAVS, Orf9b exerts its inhibitory effects on the production of IFN and proinflammatory cytokines by modulating the NF-κB signaling pathway. The N-terminal domain of the protein targets the basic NF-κB regulator NEMO, leading to the inhibition of its K63 polyubiquitination and subsequent inhibition of NF-κB activation [53].

### 3.3. The Impact of N Protein, S Protein, Orf7a, Orf3a, Nsp6, Nsp13, and Nsp14 on the JAK-STAT Pathway

To counter the potent antiviral effects generated by ISG expression, SARS-CoV-2 N protein inhibits the phosphorylation, alongside the nuclear transport of STAT1 and STAT2, which suppresses the expression of ISGs and thereby achieves immune evasion [54]. SARS-CoV-2 S protein only causes the obstruction of the STAT1 phosphorylation process by preventing the binding of JAK1 to STAT1 [41,55]. It is noteworthy that only the S1 subunit and the full-length S protein can limit the activation of ISRE promoters, while the S2 subunit does not inhibit ISRE promoter activation. This suggests that perhaps only S1 interacts with STAT1, while S2 does not participate in the attenuation of the JAK-STAT signaling pathway [41]. Orf7a has also been shown to act as a highly efficacious inhibitor of type I IFN signaling by impeding STAT2 phosphorylation through the formation of a K63 ubiquitin chain at lys119 [56,57]. Nsp6 inhibits the phosphorylation of JAK1 and TYK2, thereby preventing the formation of the STAT1/2-IRF9 complex and subsequent nuclear translocation [58].

The presence of negative regulatory factors allows proper signal transduction, but also provides opportunities for immune evasion by viruses, tumors, and others [59]. The suppressor of cytokine signaling 1 (SOCS1), a key negative regulator in the JAK-STAT pathway, is upregulated by SARS-CoV-2 Orf3a. Orf3a further exploits SOCS1 to facilitate the degradation of the JAK2 enzyme via the ubiquitin-proteasome pathway. Additionally, structural assessment of Orf3a deletion mutants indicated that the central domain of ORF3a (amino acids 70-130) is the primary determinant of upregulating SOCS1 [60]. Nsp13 and Nsp14 have the capacity to induce degradation of the type I IFN receptor within lysosomes, thereby thwarting activation of the STAT1 transcription factor, and the inhibitory effect of Nsp14 is stronger than that of Nsp13 [61].

### 3.4. Orf3a Suppresses Autophagy Activity

The mechanism that allows ORF3a to evade innate immunity is related to SNARE, a family of autophagy-associated proteins that govern the transport of substances by controlling the fusion of cell membranes. During the initiation of autophagy, a SNARE protein called STX17 is recruited to the autophagosome and forms a ternary SNARE complex with two SNARE proteins, SNAP29 and VAMP8, localized on lysosomes, promoting autophagosome–lysosome fusion and the formation of autolysosomes. After fusion, the STX17 relocates to the lysosomes [62]. This entire process requires regulation by the HOPS complex proteins, including VPS39 [63]. Orf3a can localize to endosomes/lysosomes and directly interact with the HOPS complex component VPS39. This interaction results in the sequestration of VPS39, which in turn prevents the interaction between the HOPS complex and the autophagy SNARE protein STX17, disrupting the assembly of the STX17-SNAP29-VAMP8 SNARE complex, and inhibiting the formation of autolysosomes. Additionally, Orf3a expression can also damage lysosomes, impairing their function [64,65].

The exceptional characteristics of this particular viral protein include its ability to interact with STING, thereby disrupting the STING–LC3 interaction. This, in turn, results in the obstruction of cGAS-STING-induced autophagy [66].The co-localization of STING and LC3 induced by STING activation is crucial for STING-triggered autophagy. Recent studies have shown that intracellular STING and Orf3a co-localize, and Orf3a can inhibit the co-localization of STING and LC3, indicating its ability to selectively inhibit STING-triggered autophagy to promote viral replication [66]. Following the induction of ER stress, Nsp6 is capable of binding to ER molecular chaperones HSPA5/GRP78, activating autophagy through the EIF2A/EIf2α pathway and inhibiting interferon production by promoting macrophage/autophagy-mediated STING1 degradation [67].

### 3.5. Orf6, Nsp1, and Nsp9 Block Nuclear Import and Export

SARS-CoV-2 Orf6 targets the Rae1-Nup98 complex, a component of the nuclear pore complex (NPC) cytoplasmic surface, and interacts directly with the nuclear pore component Nup98-Rae1 through its C-terminal domain, disrupting the docking of nucleoporin/importin complexes, thereby impairing the nuclear translocation of activated STAT1 [68,69,70,71]. SARS-CoV-2 Nsp1, also known as the host shutoff protein, can bind to the 40S subunit in human ribosomal complexes, inserting its C-terminal domain into the mRNA channel on the ribosome. Due to partial overlap between Nsp1 and the mRNA entry channel on the 40S subunit, it can disrupt the translation of host mRNA [72,73]. The mRNA nuclear export depends on transcription-export (TREX) complexes, where cellular mRNA binds with export factors such as the THO complex to initiate mRNA nuclear export. These export factors recruit NXF1-NXT1, facilitating mRNA transport through the nuclear pore complex (NPC) to the cytoplasm for translation. Nsp1 can prevent the correct binding of NXF1 to the mRNA export adapter and the docking of NXF1 on the nuclear pore complex [74,75]. Additionally, SARS-CoV-2 Nsp9 targets nuclear pore proteins, including Nup62, further hindering protein export and disrupting essential cellular processes [76,77].

## 4. The Evolution of Omicron to Evade Innate Immunity

As an RNA virus, SARS-CoV-2 replicates with the assistance of the RdRp enzyme, which lacks the capacity for proofreading and is therefore susceptible to the accumulating effects of mutations. The continuous accrual of mutations is markedly divergent from the original strain, ultimately leading to the emergence of a mutant strain. In comparison to other RNA viruses, SARS-CoV-2 exhibited a relatively slow mutation rate. In July 2020, the D614G mutation was identified as the first key mutation and subsequently disseminated at a rapid pace [8]. The conformational alteration resulting from the D614G mutation in the vicinity of the RBD facilitated an augmented binding affinity between the S protein and ACE2 [78]. Enhanced adaptation accelerates the dispersal of SARS-CoV-2 within the human population. By the year’s end, four new variants had emerged: Alpha (B.1.1.7), Beta (B.1.351), Gamma (P.1), and Delta (B.1.617.2) [79,80,81]. These variants were designated as variants of concern (VOC) due to their rapid spread in the initial outbreak area. Presently, these initial variants are no longer deemed to have a notable global impact on public health. The fifth VOC, Omicron, was identified in South Africa in November 2021 and soon became the dominant strain in numerous countries and regions worldwide. The main characteristics of the Omicron variant are the presence of more than 30 amino acid substitutions in the S protein and a membrane-fusion-independent entry mode [82]. There are five distinct subtypes of Omicron: BA.1, BA.2, BA.3, and BA.4/5. In the following years, the lineages of Omicron subtypes have evolved, with the emergence of the JN.1 strain, which exhibits a higher prevalence of the L455S mutation than BA.2.86. Additionally, the recombinant variant XBB.1.5, derived from BA.2.10.1 and BA.2.75, two clades of BA.2, has contributed to the ongoing transmission of Omicron subtypes, resulting in successive waves of infection [83,84].

**Figure 2:**
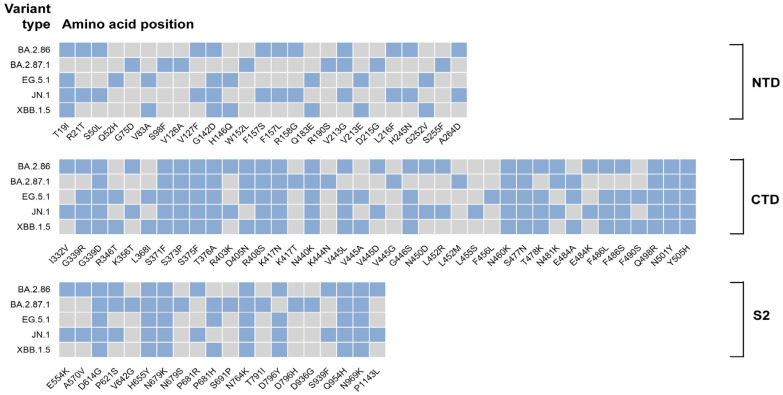
Heatmap analysis of spike protein mutation sites in recent Omicron epidemic variants BA.2.86, BA.2.87.1, EG.5.1, JN.1, and XBB.1.5. The blue squares represent mutation sites; the grey squares indicate the absence of mutations.

A phenomenal number of mutations in the S protein region of Omicron have the effect of altering the interaction with neutralizing antibodies, thereby becoming important for the escape of the adaptive immune response. However, the current evolution of SARS-CoV-2 appears to focus on small changes in the S protein region between the Omicron isoforms (Figure 2). This may imply that SARS-CoV-2 is developing ways to regulate host innate immunity through other non-spike proteins, potentially to become more adaptive to its environment. Infection-induced activation of innate immunity was markedly diminished in BA.4, BA.5, BA.2.75, and XBB.1.5, the Omicron variants, in comparison to the earlier variants BA.1 and BA.2. This observed change was associated with elevated expression of the Orf6 and N proteins [85]. As previously stated, Orf6 is responsible for immune evasion from SARS-CoV-2 through the inhibition of IRF3 phosphorylation and the nuclear translocation of STAT1. High levels of Orf6 expression prevent cells from initiating ISRE in an appropriate manner when stimulated with IFN, while a reduced IFN response favors the infection of host cells [86]. Of note, the Orf6 of BA.2 and BA.4 contains a D61L mutation, which has the effect of reducing the interaction between Orf6 and RAE1 and NUP98. Consequently, although BA.4 enhances Orf6 protein expression, it does not suppress innate immunity to the same extent as BA.5. This would also explain the evolution of Omicron in a direction that does not contain this mutation and enhances Orf6 protein levels [86,87].

The SARS-CoV-2 N protein is subject to extensive phosphorylation following infection, with the majority of these modifications occurring within a region that encompasses a serine-arginine (SR)-rich sequence [88]. It has been demonstrated that the majority of mutations observed in the N protein are concentrated within this region [89]. It is well established that phosphorylation has a profound impact on protein function, triggering structural alterations in the N protein that enhance its affinity for non-viral RNAs [90]. This may potentially influence the subsequent assembly of the virus. The N protein mutations R203K and G204R, which were previously prevalent in Alpha and Gamma mutant strains, have been reintroduced in the Omicron strain [91]. While the significance of these mutations in the N protein region remains unclear, both mutations may contribute to elevated viral loads in patients [92].

A deletion of 106–108 amino acids (ΔSGF) was apparent in the Nsp6 protein of several Omicron sublines. Although the triple amino acid deletion of BA.1 was (105–107, ΔLSG), the mutation sites essentially overlapped [93]. These amino acid changes will result in a reduction in the capacity of Nsp6 to degrade STING through autophagy, which is disadvantageous for SARS-CoV-2 to evade innate immunity. However, it is also probable that this will account for a surge in asymptomatic patients following Omicron infection and the continuation of the global epidemic [67].

## 5. Conclusions and Perspectives

**Figure 3:**
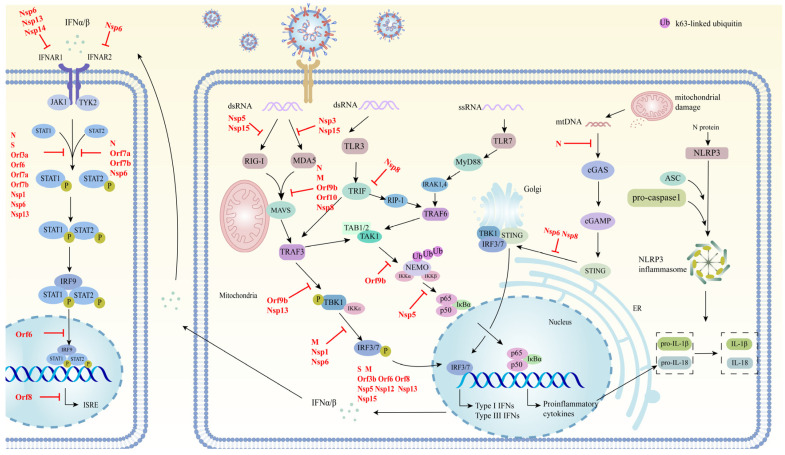
Schematic diagram of the mechanism by which SARS-CoV-2 evades innate immunity. SARS-CoV-2 proteins that target individual pathways are highlighted in red.

Innate immunity represents the primary line of defense against viral infection. The host is equipped with innate immune receptors, situated either within the cell or on the cell membrane, which enable the detection of SARS-CoV-2 and the synthesis of IFN-I, thereby instigating an immune response to combat viral infection [94]. The antiviral gene regulation of IFN exerts antiviral effects at multiple stages of the viral replication cycle, including viral attachment and entry, viral genome amplification, viral protein translation, and viral particle assembly and release [95]. In essence, if SARS-CoV-2 is to successfully replicate and spread in the host, it must evade the host’s antiviral innate immune signals. This is achieved through the encoded proteins of the virus, which act at multiple stages, including the production of IFN, signal transduction, and autophagy (Table 1) (Figure 3). Each protein may regulate a different pathway or act on the same host factor via a diverse range of mechanisms, with the overarching objective of enhancing viral replication. To illustrate, the N protein is endowed with the capacity to counteract the production of IFN by disrupting the formation of stress granules, inhibiting the ubiquitination of RIG-I, and blocking the phosphorylation of STAT1 and STAT2. Similarly, each pathway entails a synergistic action involving several proteins. The inhibition of STAT1 and STAT2 phosphorylation is facilitated by N, S, Orf7a, Orf7b, and Nsp6, whereas the M protein, Orf3b, Nsp12, and Nsp15 can block the nuclear translocation of IRF3 and damage the production and transduction of IFN. There are also some other proteins that may be involved in antagonizing the innate immune response, although the mechanisms remain unclear. For example, Mac1, one of the multiple domains of Nsp3, has been demonstrated also to help SARS-CoV-2 evade the host’s antiviral immune response [96]. Further studies of the mechanisms by which SARS-CoV-2 evades innate immunity, distinguishing whether the outcome of these escape mechanisms is to block the production of IFN or lead to autophagy, and at which stage of infection these proteins help SARS-CoV-2 to achieve innate immune escape, may assist us in searching for new antiviral strategies.

The RBD region of the SARS-CoV-2 S protein is implicated in host cell recognition and binding, and is therefore the target of the majority of neutralizing antibodies. The occurrence of amino acid substitutions within the RBD region has the potential to render neutralizing antibodies ineffective, thereby enabling the virus to evade them. Consequently, previous studies have concentrated their attention on this specific region of the spike protein. The current epidemic strains have accumulated myriad mutations in the RBD region, indicating that the S protein mutation may have reached a point of saturation [97]. The equilibrium between the capacity for cellular infection and the ability to evade immune responses is a crucial factor in the evolutionary adaptation of the virus. The growing capacity of the Omicron variant to evade the immune system, coupled with a reduction in its pathogenicity, suggests that the future evolution of SARS-CoV-2 may not be contingent on S protein mutation [98,99]. For exemplification, the Omicron variant has been observed to enhance the expression of the N protein and Orf6 [86]. However, existing studies have yet to fully elucidate the precise interaction between SARS-CoV-2, particularly the newer mutants, and the immune system. Additionally, the manner in which SARS-CoV-2 can evade the attack of innate immunity in the process of evolution remains an area that requires further investigation. It is also necessary to pay attention to the mutations of SARS-CoV-2 that can weaken its ability to evade innate immunity, and explore why such mutations occur and the role that these changes play in infection.

## Figures and Tables

**Table 1:** Overview of the mechanism of SARS-CoV-2 evasion of innate immunity.

Protein	Mechanisms	References
Nucleocapsid Protein	Dissociation with G3BP1 inhibits the cGAS-STING pathway	[25]
	Binding to E3 ubiquitin ligase TRIM25 inhibits RIG-I ubiquitination	[31,54]
	Inhibition of STAT1 and STAT2 phosphorylation	[54]
Spike Protein	Promotes the degradation of IRF3 proteasome	[40]
	Inhibits JAK1 and STAT1 binding via the S1s subunit	[41,55]
Membrane Protein	Interacts with KPNA6; inhibits IRF3 nuclear translocation	[41,51]
	Suppresses Type I Interferon Production by Ubiquitin-Mediated TBK1 Degradation	[51]
Orf3a	Blockade of autophagy activity	[64]
	Inhibits STAT1 phosphorylation by upregulating SOCS1	[60]
Orf3b	Prevents IRF3 nuclear translocation	[49,50]
Orf6	Interferes with STAT1 nuclear import by interacting with Rae1-Nup98	[68,71]
	Inhibits IRF3 nuclear translocation by interaction with KPNA2	[58]
Orf7a	Blocks STAT1 and STAT2 phosphorylation	[56,58]
	Suppresses cellular autophagy	[61]
Orf8	Hinders the interferon response through interaction with HSP90B1	[42,43,44]
Orf9b	Inhibits the interaction between TOM70 and HSP90	[34,35,36]
	Blocks NEMO’s K63 polyubiquitination	[53]
Nsp1	Interferes with cellular translation machinery	[73]
	Interacts with NXF1 to block nuclear mRNA export	[74,75]
Nsp3	Cleaves ISG15 to inhibit MDA activation	[32,33]
Nsp5	Inhibits TRIM25-mediated RIG-I ubiquitination	[38,39]
	Affects the nuclear translocation of phosphorylated IRF3	[46]
	Cleaves NEMO and TAB1	[47,48]
Nsp6	Promotes autophagy-mediated STING1 degradation and Inhibits interferon production	[67]
	Prevents STAT1 and STAT2 phosphorylation	[58]
Nsp9	Impedes nuclear export	[77]
Nsp13	Inhibits STAT1 phosphorylation	[61]
	Induces TBK1 autophagic degradation	[52,58]
	Interacts with IRF3	[45]
	Reduces the endogenous level of IFNAR1	[61]
Nsp14	Downregulates IFNAR1 expression	[61]
Nsp15	Helps viral PAMPs evade PRR recognition	[26,28,29]

## Data Availability

Not applicable.
